# Potential Application of Discarded Natural Coal Gangue for the Removal of Tetracycline Hydrochloride (TC) from an Aqueous Solution

**DOI:** 10.3390/toxics11010020

**Published:** 2022-12-26

**Authors:** Hongyou Wan, Chen Wang, Lin Gong, Xinfeng Zhu, Jingwei Yan, Jiajia Lu, Wei Zhang

**Affiliations:** 1 School of Ecology and Environment, Zhengzhou University, Zhengzhou 450001, China; 2 Research Centre of Engineering and Technology for Synergetic Control of Environmental Pollution and Carbon Emissions of Henan Province, Zhengzhou 450001, China; 3 Henan Key Laboratory of Water Pollution Control and Rehabilitation Technology, Pingdingshan 467036, China; 4 Zhengzhou Key Laboratory of Water Resource and Environment, Zhengzhou 450001, China; 5 Yellow River Institute for Ecological Protection and Regional Coordination Development, Zhengzhou University, Zhengzhou 450001, China; 6 Henan International Joint Laboratory of Water Cycle Simulation and Environmental Protection, Zhengzhou 450001, China; 7 Henan Key Laboratory of Water Resources Conservation and Intensive Utilization in the Yellow River Basin, Zhengzhou 450001, China

**Keywords:** natural coal gangue, spent coal-associated waste, removal, tetracycline hydrochloride, antibiotics pollutant, water environment

## Abstract

The generation and accumulation of discarded coal gangue (CG) have severe environmental impacts. CG can adsorb other pollutants in the aquatic environment. However, previous studies have not assessed whether CG can adsorb the emerging contaminant tetracycline hydrochloride (TC). Here, discarded CG taken from a mine was pretreated by crushing, cleaning, and sieving and subsequently applied to the adsorption of TC. The adsorption studies were carried out by batch equilibrium adsorption experiments. Our findings indicated that the adsorption behavior could be accurately described using the quasi-first order kinetic and Langmuir adsorption isotherm models, indicating that monolayer adsorption was the main mechanism mediating the interaction between CG and TC. The adsorption process was classified as a thermodynamic endothermic and spontaneous reaction, which was controlled by chemical and physical adsorption, including electrostatic interaction and cation exchange. The pH of the solution had a great influence on the TC adsorption capacity of GC, with higher adsorption occurring in acidic environments compared to alkaline environments. This was attributed to the changes in CG Zeta potential and TC pKa at different pH conditions. Collectively, our findings demonstrated the potential applicability of discarded CG for the adsorption of TC and provided insights into the adsorption mechanisms.

## 1. Introduction

The tetracycline hydrochloride (TC) is a kind of broad-spectrum antibiotic that has been widely used to treat infections in both humans and animals in recent years [1,2].

Expired TC drugs are routinely discarded into the environment and the metabolites of this antibiotic are commonly detected in sewage and wastewater due to its extensive use for medical treatment. Therefore, the levels of environmental TC have been steadily increasing [3]. The high levels of TC in aquatic environments have already been linked to adverse outcomes, including the dissemination of genes associated with bacterial antibiotic resistance, in addition to ecological damage [4]. Furthermore, the increasing occurrence of TC and related emerging pollutants in aquatic environments poses a challenge to municipal wastewater treatment [5,6].

Several methods are commonly used to remove antibiotic pollution from wastewater such as biological processing technology [7], catalytic degradation [8], photocatalytic degradation [9], and adsorption [10,11]. Among these, adsorption is a promising alternative for the removal of emerging pollutants from water environments due to its effectiveness, low cost [12,13], and ease of implementation, in addition to producing fewer by-products than other methods. Several recent studies have assessed the applicability of many kinds of adsorbents for the removal of TC or related emerging pollutants from aqueous solutions, including ferrihydrite and goethite [14], carbon materials [15], biochar [16], and carbon nanotubes [17]. The main adsorption mechanisms of antibiotic pollutants in water environments are physical adsorption, chemical adsorption, and electrostatic interaction [18,19,20]. As previously reported, modified attapulgite could efficiently remove TC from water environment [21]. Bentonite could also efficiently remove ciprofloxacin hydrochloride (CIP) from aqueous solutions [22]. The potential applications of CG in the removal of traditional pollutants including phosphates [23], metal ions (Cu^2+^, Co^2+^) [24], and F^−^ [25] have been investigated. However, no previous studies have assessed the applicability of discarded CG as an adsorbent material without chemical modification to remove hydrochloride (TC) or related emerging pollutants from an aqueous solution. Natural CG is a widespread by-product generated in the process of coal mining or the washing process in the coal industry [26]. CG is usually a black-gray rock with abundant minerals, organic substances, and stable crystals [27,28]. However, the accumulation of excessive CG in coal related operations can lead to groundwater, air, and land pollution [29,30], and therefore it is crucial to explore new methods for the recycling of CG materials. Natural CG could be used to efficiently remove TC from aqueous solutions due to its high abundance, low cost, high chemical stability, and high ion exchange capacity [13,31].

In this study, the applicability of discarded natural CG for in the removal of TC from an aqueous solution was systematically investigated. The effect of CG dosage, CG particle size, TC concentration, contact time, temperature, and solution pH on TC adsorption by CG were studied. Additionally, adsorption kinetics and adsorption isotherm fitting were performed to interpret the adsorption data. Material characterization routes including FT-IR, XPS, XRD, and SEM were applied to analyze the structural changes of the CG in the adsorption process. This study thus provides innovative insights into the potential applicability of spent CG derived from coal-related waste for the efficient removal of antibiotic contaminants from water.

## 2. Materials and Methods

### 2.1. Raw Material and Chemicals

**Figure 1:**
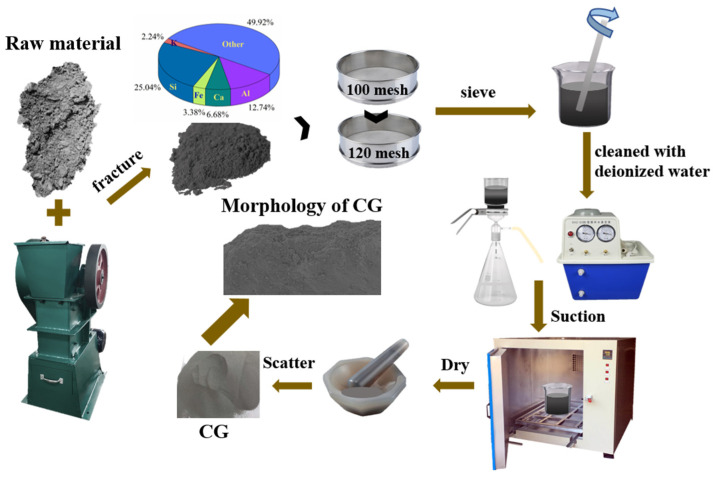
The flow chart for treatment of CG samples derived from the discarded natural coal gangue.

The raw CG (coal gangue) material was directly collected from the fifth mine of Pingdingshan Tian’an Coal Mining in the city of Pingdingshan, Henan Province, China. The treatment process of discarded CG as adsorbate was briefly depicted in Figure 1. The CG samples were firstly dried under 45 °C in a thermostatic oven for 12 h, fractured by jaw breaker, and then finally sieved through the first 100-mesh sieve and the second 120-mesh sieve. The CG samples (with particle-size range of 0.12 to 0.15 mm) were first cleaned with deionized water, filtered by vacuum sucking pump, and then dried at 40 °C in thermostatic oven for 5 h. The CG samples were finally grinded to disperse, and then stored for further use. The elemental compositions of Si, Al, Ca, Fe, and K in the raw CG were determined as 25.0%, 12.7%, 6.7%, 3.4%, and 2.4%, respectively (Table S1). The TC (tetracycline, C_22_H_25_ClN_2_O_8_, 96%) was bought from Aladdin Biochemical Co., Ltd., Shanghai, China. The NaOH (sodium hydroxide, 96%) was obtained from Tianjin Sailboat Chemical Reagent Technology Co., Ltd., Tianjin, China, while the H_2_SO_4_ (sulfuric acid, 98%) was bought from Luoyang Chemical Reagent Factory, Henan, China.

### 2.2. Adsorption of TC by CG

**Figure 2:**
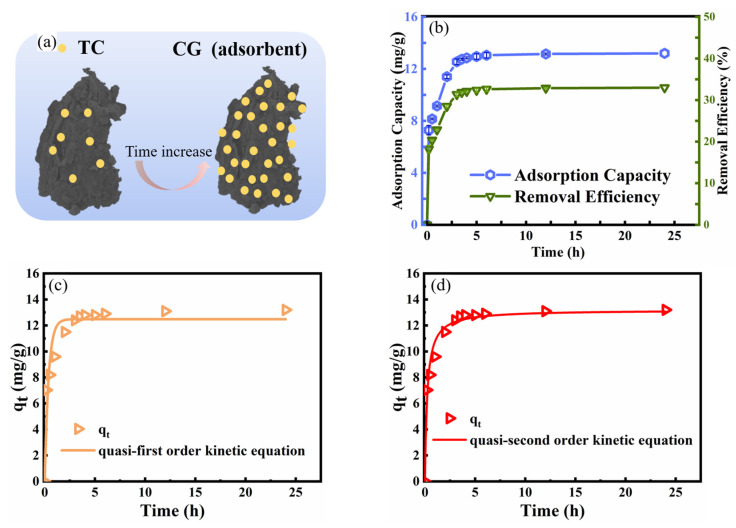
The adsorption kinetic curves of TC by CG: (**a**) the proposed schematic diagram of TC adsorption by CG with increasing contact time; (**b**) effect of contact time on TC adsorption by CG, (**c**) the quasi-first order kinetic model fitting line; (**d**) the quasi-second order kinetic model fitting line. (TC concentration of 40 mg/L, CG dosage of 1 g/L, pH of 6.2, contact temperature of 25 °C, and stirring speed of 120 rpm).

The stock TC solution (40 mg/L) was prepared by weighing 20 mg TC into a 100 mL beaker, adding a small amount of water to dissolve and pipette into a 500 mL volumetric flask, rinsing the beaker multiple times to pipette, and fixing the volumetric flask to the 500 mL tick line. For each batch experiment, the TC solution (50 mL, 40 mg/L) was mixed with CG (1 g/L) in the conical flask (100 mL) shaking by the thermostatic water bath oscillator (120 rpm) at 298 K until adsorption equilibrium was reached. At the terminal of 3 h, the slurry containing the CG particles and TC was quickly separated through a 0.45-μm syringe filter to get the filtrate. The filtrate after separation was measured by using an Ultraviolet–visible spectrophotometer (UV759CRT, Shanghai, China) at a wavenumber of 357 nm and the proposed process for adsorption of TC by CG was shown in Figure 2a. The ICP (inductively coupled plasma-atomic emission spectroscopy, AAS, AA800, Perkin-Elmer) was used to measure the concentration of iron leaching from CG into the solution during the TC adsorption process. The TC desorption from exhausted CG and CG recycling process were conducted (Texts S1 and S2).

The adsorption capacity (*Q*, mg/g) and removal efficiency (*E*, %) of TC by CG were calculated by Equations (1) and (2), respectively.

(1) $$Q=\left[\frac{C_{0}-C}{m}\right]\times V$$

(2) $$E=\left[\frac{C_{0}-C}{C_{0}}\right]\times 100\%\;\;$$

where *C*_0_ and *C* (mg/L) represent the initial and equilibrium concentration of TC during the adsorption process, respectively; *m* (g) is the dosage of CG; *V* (L) is the volume of TC solution.

#### 2.2.1. Adsorption Kinetics and Isotherms Investigation

In the adsorption kinetics investigation experiments, the slurry of TC solution (50 mL, 40 mg/L) and CG (1 g/L) was mixed in conical flasks (100 mL) by the water bath oscillator at 298 K. The pH of the slurry determined as 6.2 without pH adjustment. After the beginning of the reaction, at given time intervals of 0.17, 0.5, 1, 2, 3, 3.5, 4, 5, 6, 12, and 24 h, the slurry (3 mL) was withdrawn from each conical flask to measure the concentration of TC.

Aiming to analyze the adsorption kinetics of TC by CG particles, the quasi-first-order Equation (3) and quasi-second order kinetic Equation (4) were used to explain adsorption kinetics.

(3) $$q_{t}=q_{e}\left(1-e^{-k_{1}t}\right)$$

(4) $$q_{t}=\frac{k_{2}q_{e}^{2}t}{1+k_{2}q_{e}t}$$

where *q_e_* (mg/g) is the amount of TC adsorbed by CG at equilibrium and *q_t_* is the adsorption amount at certain time of *t*. The *k*_1_ (h^−1^) and *k*_2_ (h^−1^) represent the first order rate constant and the second order rate constant, respectively [32,33].

The adsorption isotherms investigations were conducted by measuring the adsorption capacities of TC by CG at different temperatures of 298 K, 308 K, and 318 K. At the three-mentioned temperatures, three parallel samples (for each temperature) were prepared by mixing TC (50 mL, 20–150 mg/L) solution and CG (1 g/L) into the conical flasks (100 mL) in the water bath oscillators (with oscillating speed of 120 rpm) for 3 h. All samples were conducted in triplicate. At the termination of the setting adsorption equilibrium time, the solution samples were quickly withdrawn, then filtered through a 0.45-μm syringe filter, and finally measured by UV–Vis spectrophotometry.

The Langmuir and Freundlich isotherm models for different temperature (25 °C, 35 °C, 45 °C) adsorption of TC were applied to assess adsorption process.

(5) $$Q=\frac{Q_{m}C_{e}K_{L}}{1+K_{L}C_{e}}$$

(6) $$Q=K_{F}C_{e}^{\frac{1}{n}}$$

where *Q* is the adsorption amount at equilibrium time (mg/g), *Q_m_* is the maximum adsorption capacity at a certain temperature (mg/g), *C_e_* is the concentration of adsorbate in solution at adsorption equilibrium (mg/L), *K_L_* demonstrates Langmuir adsorption equilibrium constant (mg/g), and *K_F_* is Freundlich adsorption equilibrium constant (mg/g), the value of 1/*n* represents an adsorption intensity [34,35].

#### 2.2.2. Effect of CG Particle Size

The effects of CG particle sizes on adsorption performance of TC by CG were performed at ranging particle sizes (0.90–0.45, 0.45–0.30, 0.30–0.15, 0.15–0.12, and ≤0.12 mm). The CG (with different sizes) samples (1 g/L) were mixed with TC solution (50 mL, 40 mg/L) without adjusting the solution pH of 6.2 in the conical flask (100 mL) in the water bath oscillator (120 rpm). The contact time and temperature were 3 h and 298 K, respectively.

#### 2.2.3. Effect of CG Dosage and pH

The effects of CG dosage on TC adsorption were explored at the CG dosage (size of 0.15–0.12 mm) ranged of 0 and 2 g/L, a natural pH of 6.2, and temperature of 298 K under 120 rpm in water bath oscillator. The volume of TC solution (40 mg/L) was 50 mL, with contact time of 3 h. Finally, the effect of pH on the adsorption of TC by CG was investigated in pH ranging from 2 to 12 at the CG dosage of 1 g/L for 3 h. The contact temperature was 298 K in the water bath oscillator (120 rpm).

### 2.3. Material Characterization

The XRD pattern of CG was obtained by using the D/max 2500 pc (Rigaku, Empyrean, The Netherlands) between the 2θ of 5–90°. The elements composition of the original CG was measured by the XRF (X-ray Fluorescence Spectrometer, ARL, Thermo Scientific, Waltham, MA, USA). The FT-IR spectra (Fourier transform infrared spectroscopy, Vertex 80, Bruker, Germany) was used to investigate the key functional groups in CG before and after the adsorption process experiment. The XPS (X-ray photoelectron spectroscopy, Escalab 250Xi, Thermo Scientific, Waltham, MA, USA) instrument was used to measure the chemical state of main elements of CG. The Zeta potentials of CG in aqueous solution were recorded through a micro-electrophoresis instrument (JS94HM, Shanghai, China). The SEM (scanning electron microscope, Merlin compact, Zeiss, GER) was used to analyze the morphology of CG. The N_2_-BET measurement (Brunauer–Emmett–Teller, ASAP 2460, Micromeritics, Norcross, GA, USA) was used to analyze the surface area of CG samples, while the BJH (Barrett–Joyner–Halenda) was used to obtain the pore volume and pore size of CG under N_2_–adsorption/desorption process.

## 3. Results

### 3.1. Adsorption Kinetic Study

The effect of the reaction time on TC adsorption by CG is shown in Figure 2b. Approximately 33.2% of TC was removed by CG within 24 h, with a TC adsorption capacity by CG of 13.3 mg/g. The TC adsorption capacity by CG was quickly increased within 0–3 h, whereas the TC adsorption capacity was nearly constant within 12–24 h. The quasi-first order equation and quasi-second order kinetic equations were used to fit the TC adsorption kinetic results. The fitting curves (Figure 2c,d) and their detailed parameters (Table S2) indicated that the quasi-second order kinetic equation could better describe the adsorption of TC by CG compared with the quasi-first order equation. Furthermore, the *q_e_* (13.19 mg/g) calculated by the quasi-second order kinetic equation was close to the experimental adsorption capacity (12.74 mg/g), further confirming the suitability of the quasi-second order kinetic equation to fit the TC adsorption by CG in the water environment. The quasi-second order kinetic equation could further indicate that the adsorption performance of TC by CG should be controlled by chemical adsorption [36].

### 3.2. Adsorption Isotherms Study

**Figure 3:**
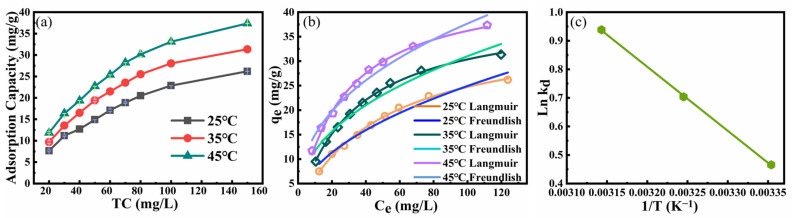
The adsorption isotherms curves of TC by CG: (**a**) impact of initial TC concentration on adsorption capacity under different temperatures (25 °C, 35 °C, 45 °C), (CG dosage of 1 g/L, contact time of 3h, pH of 6.2 and 120 rpm); (**b**) Langmuir and Freundlich adsorption isotherm of TC adsorbed by CG under different temperature (25 °C, 35 °C, 45 °C), (**c**) Plot of *LnK_d_* vs. 1/T for TC adsorption by CG.

The effect of TC concentration on the TC adsorption performance by CG was illustrated in Figure 3a. With the TC concentration ranging from 20 mg/L to 150 mg/L, the TC adsorption capacity was increased from 7.2 mg/g to 24.2 mg/g. With the increasing TC molecules in an aqueous solution, the unit mass of CG would contact and adsorb more TC molecules, thus increasing the adsorption capacity of TC by CG [37]. However, the TC adsorption efficiency was decreased from 36.1% to 16.1% with TC concentration ranging from 20 mg/L to 150 mg/L. The decrease in TC adsorption efficiency was mainly attributed to the adsorption saturation of TC by the limited CG samples in the water environment. With more TC molecules added to the solution, the calculated TC adsorption efficiency by CG would be decreased due to the increased C_0_ in Equation (2) [38].

The effect of contact temperature (25 °C, 35 °C, and 45 °C) on TC adsorption by CG is shown in Figure 3a. As the contact temperature increased, the adsorption capacity of TC by CG was increased, indicating that the adsorption of TC by CG was an endothermic process [39].

The fitting curves for adsorption isotherms of TC by CG and their related parameters are shown in Figure 3b and Table S2, respectively. Based on the fitting curves at 25 °C, the Langmuir mode (with a correlation coefficient of 0.997) could better describe the TC adsorption by CG, compared with the Freundlich model (with a correlation coefficient of 0.972). These results confirmed that the adsorption of TC by CG was mainly controlled by monolayer adsorption [40].

The thermodynamic parameters, including Δ*G*, Δ*H*, and Δ*S*, were described by Equations (7), (8), and (9), respectively.

(7) $$LnK_{d}=\frac{q_{e}}{c_{e}}$$

(8) $$\Delta G=-RTLnK_{d}$$

(9) $$LnK_{d}=\frac{\Delta S}{R}-\frac{\Delta H}{RT}$$

where Δ*G* (J/mol) is the Gibbs free energy; Δ*H* (KJ/mol) is the enthalpy change of the adsorption process; Δ*S* (J/mol·K) is the entropy change between product and reactant; *R* is the thermodynamic constant, which is approximately equal to 8.314 (J/mol·K); *T* (K) is the thermodynamic temperature. The values of Δ*S* and Δ*H* are computed from the intercept and the slope of the linear plot for *LnK_d_* vs. 1/T [41], which is shown in Figure 3c. The values of Δ*G* can be calculated from Equation (8). The results are displayed in Table 1.

The positive Δ*H* (18.63 KJ/mol) (Table 1) further confirmed that the TC adsorption by CG was an endothermic process. The negative values of Δ*G* indicated that the adsorption process was spontaneous. The positive Δ*S* value (66.34 J/mol·K) indicated that the freedom and randomness of the adsorption process were higher [39,41,42].

### 3.3. Effect of CG Dosage and Particle Size

**Figure 4:**
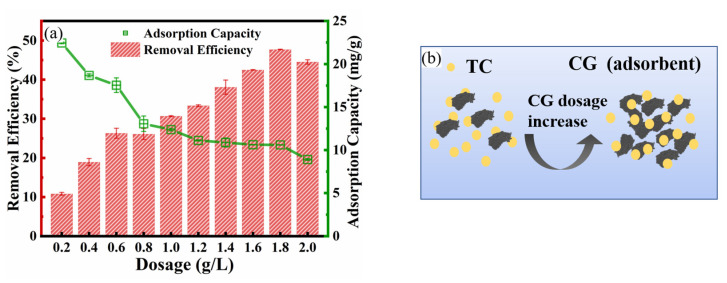
Effect of adsorbent dosage for TC adsorption: (**a**) Effect of CG dosage on removal efficiency and adsorption capacity of TC by CG (contact time of 3 h, TC concentration of 40 mg/L, pH of 6.2 and temperature of 25 °C, 120 rpm); (**b**) the proposed schematic diagram of TC adsorption by CG with increasing CG dosage.

Figure 4a illustrates the effect of CG dosage on TC adsorption by CG. With CG dosage ranging from 0.2 g/L to 1.8 g/L, the TC adsorption efficiency increased from 10.8% to 47.7%. The enhancement of TC adsorption efficiency with increasing CG dosage was mainly attributed to the increase in the number of adsorption sites provided by more CG particles (Figure 4b). However, the adsorption efficiency of CG at a dosage of 2 g/L (44.54%) is slightly lower than at a dosage of 1.8 g/L (47.72%), which was mainly due to the insufficient exposure of CG active sites caused by the agglomeration of CG particles [43,44]. However, The TC adsorption capacity was decreased from 22.6 mg/g to 8.79 mg/g with CG dosage ranging from 0.2 g/L to 2 g/L. After increasing the CG in the aqueous solution, more CG particles cannot adsorb the TC (due to the limited TC concentration). Thus, the calculated adsorption capacity was decreased due to the increased m of Equation (1).

**Figure 5:**
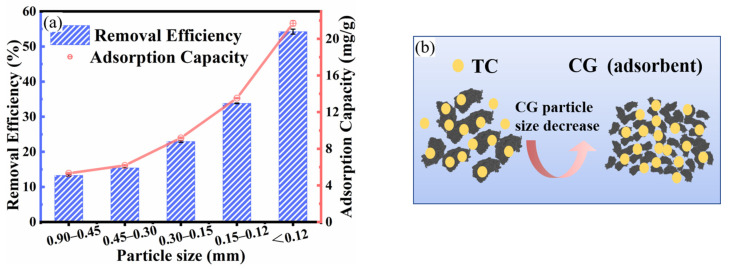
Effect of adsorbent particle size for TC adsorption: (**a**) Effect of CG particle size on removal efficiency and adsorption capacity of TC by CG (contact time of 3 h, TC concentration of 40 mg/L, CG dosage of 1 g/L, pH of 6.2 and temperature of 25 °C, 120 rpm), (**b**) the proposed schematic diagram of TC adsorption by CG with reduced CG particle size.

The TC adsorption efficiency was increased from 13.30% to 54.26% with CG (1 g/L) particle size ranging from 0.90–0.45 mm to smaller than 0.12 mm (Figure 5a). The CG with a smaller particle size would provide more specific surface areas to adsorb TC in an aqueous solution, further increasing the TC adsorption efficiency, which is depicted in Figure 5b.

### 3.4. Effect of pH

**Figure 6:**
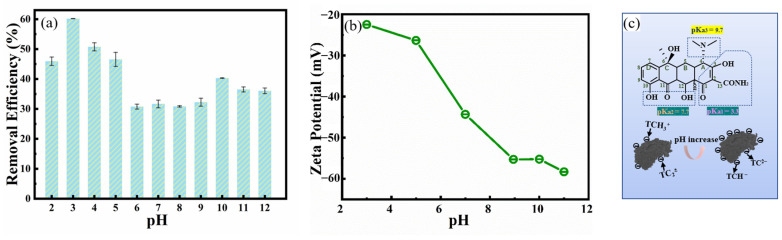
Effect of solution pH for TC adsorption: (**a**) Effect of solution pH on removal efficiency of TC by CG (contact time of 3 h, TC concentration of 40 mg/L, CG dosage of 1 g/L, contact temperature of 25 °C, and stirring speed of 120 rpm); (**b**) the Zeta potential of CG with increasing pH in an aqueous solution, (**c**) the proposed schematic diagram of TC adsorption by CG with increasing solution pHs.

The effect of pH on TC adsorption by CG in an aqueous solution was shown in Figure 6a. The TC adsorption efficiency by CG in an acidic environment was higher compared with the more alkaline environment [45]. The significant difference in the adsorption efficiency in different pH conditions could be explained by the Zeta potential data (Figure 6b). The dominant phase of TC in the aqueous solution was mainly TCH^3+^ cation (pH < 3), TC^2±^ zwitterion species (pH between 3.3 and 7.7), and TCH^−^/TC^2−^ (pH > 7.7) [46]. Therefore, the TC molecules were mainly positively charged. With the pH of solution changing from acidic to alkaline, the charge of CG was becoming more negative. Thus, compared with the acidic environment, the electrical repulsion between the CG and TC would be increased in the more alkaline environment, leading to decreased TC adsorption capacity by CG (Figure 6c) [46,47].

### 3.5. TC Desorption and the Reusability of Exhausted CG

The TC desorption rate from exhausted CG was shown in Figure S1, while TC desorption rate from exhausted CG reached only 17.6% at equilibrium time of 1.5 h. This result indicated TC were not easily desorbed from CG, further confirming the relatively stronger force between CG and TC [48,49].

The reusability of exhausted CG in TC removal process was conducted and the TC removal efficiency by used CG could only reach 25.3%. This result illustrated that the exhausted CG was needed further treatment before its reusability.

### 3.6. Material Characterization of the Natural CG and its Adsorption Mechanism

**Figure 7:**
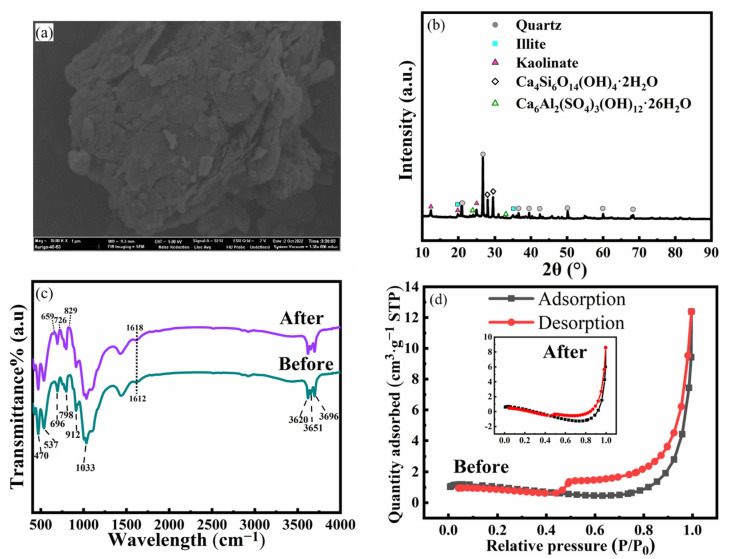
The material characterization results of CG particles: (**a**) SEM images of the CG before adsorption; (**b**) XRD; (**c**) The FT-IR spectra of the CG before and after adsorption; (**d**) the nitrogen adsorption–desorption isotherms before and after reaction.

The SEM image of CG is shown in Figure 7a. The surface of CG is flat and accompanied by flaky kaolin stones, showing a cluttered surface with an irregular arrangement. The XRD patterns of the original CG are shown in Figure 7b. The quartz, kaolinite, quartz, illite, ettringite (Ca_6_Al_2_(SO_4_)_3_(OH)_12_·26H_2_O), and C–S–H (Ca_4_Si_6_O_14_(OH)_4_·2H_2_O) are the dominant crystal phases in the CG [50]. The chemical state of the main elements in CG was investigated and shown in Figure S2.

The detailed BET parameters of CG were shown in Table S3. As depicted in Figure 7d, the isotherm curves are fitted with the IUPAC classification type curve of IV and H_3_ type hysteresis loop. Meanwhile, the hysteresis loop closes at a relative pressure of P/P_0_ = 0.4, indicating the existence of smaller mesopores in the CG sample and a smaller amount of micropores [51,52]. The BET surface area and the total pore volume of CG after the TC adsorptions processes were decreased, and the reasons are illustrated as follows: (1) TC enters the pores of the material and stays in the pores by electrostatic attraction. (2) The active group in the material undergoes ion exchange or complexation reaction with TC, thereby reducing the pore volume and specific surface area.

The FTIR spectra of CG before and after the TC removal process is shown in Figure 7c. The characteristic bands of CG observed at 3696, 3651, and 3620 cm^−1^ are mainly attributed to the hydroxyl stretching vibrations of kaolinite. The peak observed at 470 cm^−1^ is related to the Si-O-Fe, further confirming the existence of Fe. The peaks observed at 694 cm^−1^ and 537 cm^−1^ were representative of the stretching and bending vibrations of Si-O-Al, respectively. The band observed at 912 cm^−1^ is related to the Al-OH bending vibration. The peaks observed at 798 cm^−1^ and 1033 cm^−1^ are mainly attributed to the bending vibration and stretching vibration of Si-O-Si in the natural CG, which are identical with the characteristic absorption peaks of illite, indicating the existence of illite and kaolinite in CG, according to the XRD results [53,54,55,56,57].

The FT-IR spectra show characteristic bands of original CG before and after TC adsorption. Compared with the CG before adsorption, the -OH in absorption peaks at 3651, and 3620 cm^−1^ of CG after adsorption had almost no change. With the peak at 912 cm^−1^ of Al-OH absorption peak weakened after adsorption, suggesting that the decrease in Al-OH may be due to the complexation between Al and TC. The decrease of Si-O-Al absorption peaks at 537 cm^−1^ indicates the damage of kaolinite. This, accompanied by the strengthening of Si-O-Si bending vibration peaks at 798 cm^−1^ and the lower Si-O-Si stretching vibration peaks at 1033 cm^−1^, indicated the destruction of illite. Nevertheless, the peaks at 659, 726, and 829 cm^−1^ present varying degrees of elevation due to the consequence of TC adsorption onto CG, which represented the characteristic peaks of TC [55,58,59,60]. Furthermore, the peak observed at 1612 cm^−1^ was mainly attributed to the vibration of amide carbonyl at ring A of the TC (Figure 6c). Therefore, the narrower and higher intensity peak observed at 1618 cm^−1^ of CG after TC adsorption could confirm the deprotonation of TC during the adsorption process [61]. Figure S2 could also support the deprotonation of TC.

**Figure 8:**
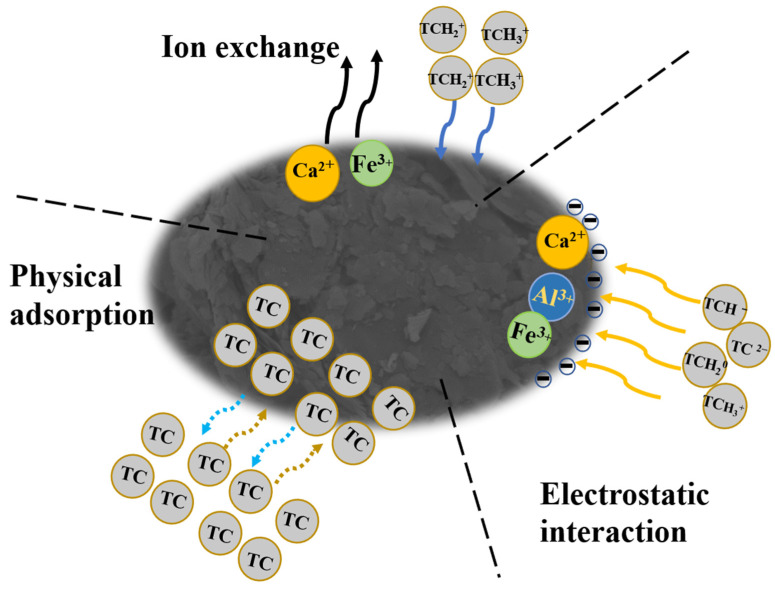
The proposed adsorption mechanism of TC adsorption by CG.

Based on the above analysis, the possible adsorption mechanism of TC by CG is shown in Figure 8. The Langmuir model of the TC adsorption process indicated that the physical adsorption process will also work in the TC adsorption by CG. The electrostatic action between TC and CG at varying pH would also affect the adsorption of TC by CG in an aqueous solution [62].

Additionally, the adsorption kinetic analyses confirmed that the adsorption of TC by CG was affected by the chemical adsorption process. Considering that the TC was an amphoteric molecule [46], the TC would occur in the protonation and deprotonation reactions at varying pH conditions. At a pH lower than pK_a1_ (3.3), the TC was dominant with species of TCH^3+^. With the pH ranging between pK_a1_ and pK_a2_ (3.3 < pH < 7.7), the TC was mainly presented with TCH^2+^. At pH further increasing to a range of (7.7 < pH < 9.7) and (pH > 9.7), the dominant species of TC were TCH^−^ and TC^2−^, respectively [47]. The species of TCH^3+^ and TCH^2+^ in acidic and neutral environments would be exchanged with the positively charged ions (Ca^2+^, Fe^3+^) in CG. Additionally, the concentrations of Fe and Ca were determined as 140.8 µg/L and 173.2 µg/L, respectively, further confirming the metal element exchange from the CG to the TC solution [63,64]. Therefore, at acidic and neutral pH environments, the cation exchange process would affect the adsorption of TC by the CG samples. However, in the alkaline pH environment, the cation exchange between the TCH^–^/ TC^2−^ and CG would weaken.

The comparisons of TC adsorption performance by CG and other related minerals were shown in Table 2. Compared with kaolinite [64] and ferrihydrite [14], the natural CG had a higher TC adsorption capacity and a shorter adsorption equilibrium time (3 h). Compared with minerals including, such as illite [65], montmorillonite [62], and bentonite clay [14], natural CG has a lower TC adsorption capacity but reaches adsorption equilibrium within a shorter time of 3 h [66,67,68].

Based on the aforementioned comparison, the CGs used in this study possessed several advantages including a faster equilibrium time, relatively larger adsorption capacity, and easier access to application.

## 4. Conclusions

In the present study, CG was prepared by simple pretreatment for the removal of TC in solution. The effects of contact time, the particle size of CG, the dosage of CG, different temperatures, and solution pH on the adsorption capacity were analyzed. The adsorption experimental data can be well fitted by the first-order kinetic model and Langmuir adsorption isotherm model. The results showed that the adsorption process can be classified as spontaneous endothermic monolayer adsorption, controlled by physical adsorption and chemical adsorption. The adsorption model, FT-IR, XPS, XRD, and Zeta potential show that the adsorption mechanism of TC by CG mainly includes electrostatic interaction and cation exchange. In addition, the strength of electrostatic attraction is the main reason for the difference in adsorption capacity between acidic and alkaline environments. The comparison between CG and other minerals showed that CG had better adsorption abilities for TC. This study confirmed that CG is a promising and sustainable adsorbent that could be used for the treatment of antibiotic-contaminated wastewater. Therefore, CG can be used to both realize the waste resource reduction of CG and explore the enhanced value of CG in wastewater treatment.

## Figures and Tables

**Table 1:** The thermodynamic parameters for TC adsorption by CG.

**T (K)**	**ΔG (J/mol)**	**ΔH (KJ/mol)**	**ΔS (J/mol·K)**	**R^2^**
298	−1154			
308	−1744	18.635	66.347	0.999
318	−2326			

**Table 2:** The comparisons of TC adsorption between CG and other minerals.

Used Minerals	Concentration of Pollutants and Adsorption Equilibrium Time	Adsorption Capacity Q_max_ (mg·g^−1^)	References
Natural CG (1 g/L)	TC (40 mg/L), 3 h	24.7 mg/g	This Study
bentonite clays (0.4 g/L)	TC (30 mg/L), 5 h	156.7 mg/g	[11]
Ferrihydrite (10 g/L)	TC (40 mg/L), 96 h	3.2 mg/g	[14]
Montmorillonite (0.2 g/L)	TC (5–100 mg/L), 24 h	250.0 mg/g	[60]
Kaolinite (100 g/L)	TC (480 mg/L), 8 h	4.4 mg/g	[62]
Illite (10 g/L)	TC (200 mg/L), 8 h	32.0 mg/g	[63]

## Data Availability

Not applicable.

## Supplementary Materials

The following supporting information can be downloaded at: https://www.mdpi.com/article/10.3390/toxics11010020/s1, Text S1: TC desorption from the exhausted CG; Text S2: The CG recycling process and the results; Table S1: The elemental compositions of the raw CG; Table S2: The detailed parameters for adsorption kinetics and isotherms of TC by CG; Table S3: BET parameters of CG before and after TC adsorption; Figure S1: The desorption for TC of exhausted CG; Figure S2: The XPS spectra of the raw and used CG.
