# Supplementary material for: Potential Application of Discarded Natural Coal Gangue for the Removal of Tetracycline Hydrochloride (TC) from an Aqueous Solution
Source: Toxics. 2022 Dec 26;11(1):20. doi: 10.3390/toxics11010020 (PMC9865974; doi:10.3390/toxics11010020)
Supplement: Supplementary file 1 [file toxics-11-00020-s001.zip › toxics-2059505-supplementary.pdf]

toxics

Supplementary Materials

Potential Application of Discarded Natural Coal Gangue for the Removal of Tetracycline Hydrochloride (TC) from an Aqueous Solution

Hongyou Wan [1,2], Chen Wang [1], Lin Gong [1], Xinfeng Zhu [3], Jingwei Yan [1], Jiajia Lu [1] and Wei Zhang [1,2,3,4,5,6,7,*]

1 School of Ecology and Environment, Zhengzhou University, Zhengzhou 450001, China
2 Research Centre of Engineering and Technology for Synergetic Control of Environmental Pollution and Carbon Emissions of Henan Province, Zhengzhou 450001, China
3 Henan Key Laboratory of Water Pollution Control and Rehabilitation Technology, Pingdingshan 467036, China
4 Zhengzhou Key Laboratory of Water Resource and Environment, Zhengzhou 450001, China
5 Yellow River Institute for Ecological Protection and Regional Coordination Development, Zhengzhou University, Zhengzhou 450001, China
6 Henan International Joint Laboratory of Water Cycle Simulation and Environmental Protection, Zhengzhou 450001, China
7 Henan Key Laboratory of Water Resources Conservation and Intensive Utilization in the Yellow River Basin, Zhengzhou 450001, China
* Correspondence: zhangwei88@zzu.edu.cn

Number of pages: 5

Number of Texts: 2

Number of Tables: 3

Number of Figures: 2

Supplementary captions

Text S1 TC desorption from the exhausted CG

Text S2 The CG recycling process and the results

Table S1 The elemental compositions of the raw CG

Table S2 The detailed parameters for adsorption kinetics and isotherms model of TC by CG

Table S3 BET parameters of CG before and after TC adsorption.

Figure S1 The desorption for TC of exhausted CG.

Figure S2 The XPS spectra of the raw and used CG.

Text S1 TC desorption from the exhausted CG

The used CG after TC adsorption process was firstly filtered and dried under natural conditions for 3 h. Then, the used CG (1 g/L) was added into deionized water in the erlenmeyer flask (100 mL). The slurry was mixed by water bath oscillator (120 rpm and 25℃), and the samples (3 mL) was withdrawn from the slurry at time intervals of 0.17, 0.5, 1, 2, 3, 3.5, 4, 5, 6, 12, and 24 h. The withdrawn samples were quickly filtered and measured the released TC concentrations.

Text S2 The CG recycling process and the results

The used CG after TC adsorption process was firstly filtered and dried under natural conditions for 3 h. Then, the used CG (1 g/L) was added into TC (40 mg/L, 50 mL) in the erlenmeyer flask (100 mL). The slurry was mixed by water bath oscillator (120 rpm and 25℃), and the samples (3 mL) was withdrawn from the slurry at equilibrate time of 3h. The withdrawn samples were quickly filtered and measured the residual TC concentrations.

Table S1 The elemental compositions of the raw CG

Elements	Elements Content (%)
Al	12.7419
As	0.0037
Ba	0.0906
Ca	6.6808
Cl	0.0670
Cr	0.0479
Cu	0.0060
Fe	3.3760
Ga	0.0030
K	2.2417
Mg	0.8522
Mn	0.0471
Na	0.4297
Ni	0.0246
P	0.0557
Rb	0.0103

Si	25.0436
S	0.7195
Sr	0.0523
Ti	0.6653
Zn	0.0170
Zr	0.0200

Table S2 The detailed parameters for adsorption kinetics and isotherms of TC by CG.

		Parameter 1	Parameter 2	R^2
Adsorption kinetics	Quasi first order kinetic equation	K_1=2.6366（1/h）	Q_e=12.48 (mg/g)	0.919
	Quasi second order kinetic equation	K_2=0.3372 (g/(mg·h))	Q_e=13.194(mg/g)	0.973
Adsorption isotherms (25℃)	Langmuir	K_L=0.01998(L/mg)	Q_m=37.106 (mg/g)	0.997
	Freundlich	K_F=2.611($mg^{(1-n)}$ ·L^n/g)	n=2.04	0.972
Adsorption isotherms (35℃)	Langmuir	K_L=0.02985(L/mg)	Q_m=40.540 (mg/g)	0.998
	Freundlich	K_F=4.375 ($mg^{(1-n)}$ ·L^n/g)	n=2.35	0.957
Adsorption isotherms (45℃)	Langmuir	K_L=0.03769(L/mg)	Q_m=45.800(mg/g)	0.995
	Freundlich	K_F=5.927($mg^{(1-n)}$ ·L^n/g)	n=2.15	0.972

Table S3 BET parameters of CG before and after TC adsorption.

Properties	Samples	
	Before TC adsorption	After TC adsorption
BET Surface Area (m^2 g^{-1})	5	2
Vm (cm^3(STP) g^{-1})	1.63*10^{-3}	6.00*10^{-4}
Total pore volume (cm^3 g^{-1})	19.16*10^{-3}	13.28*10^{-3}
Mean pore diameter (nm)	15.48	18.57
Langmuir surface area (m^2 g^{-1})	4.23	0.99
BJH surface area (m^2 g^{-1})	1.78	0.65

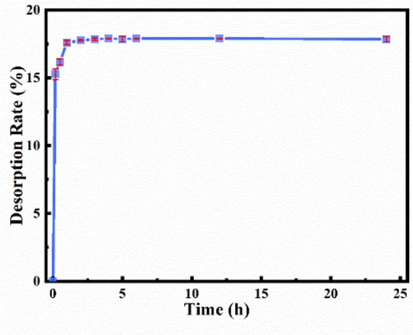

Figure S1. The desorption for TC of exhausted CG (CG dosage of 1 g/L, pH of 7.0, contact temperature of 25 °C, deionized water volume of 50 mL, and stirring speed of 120 rpm).

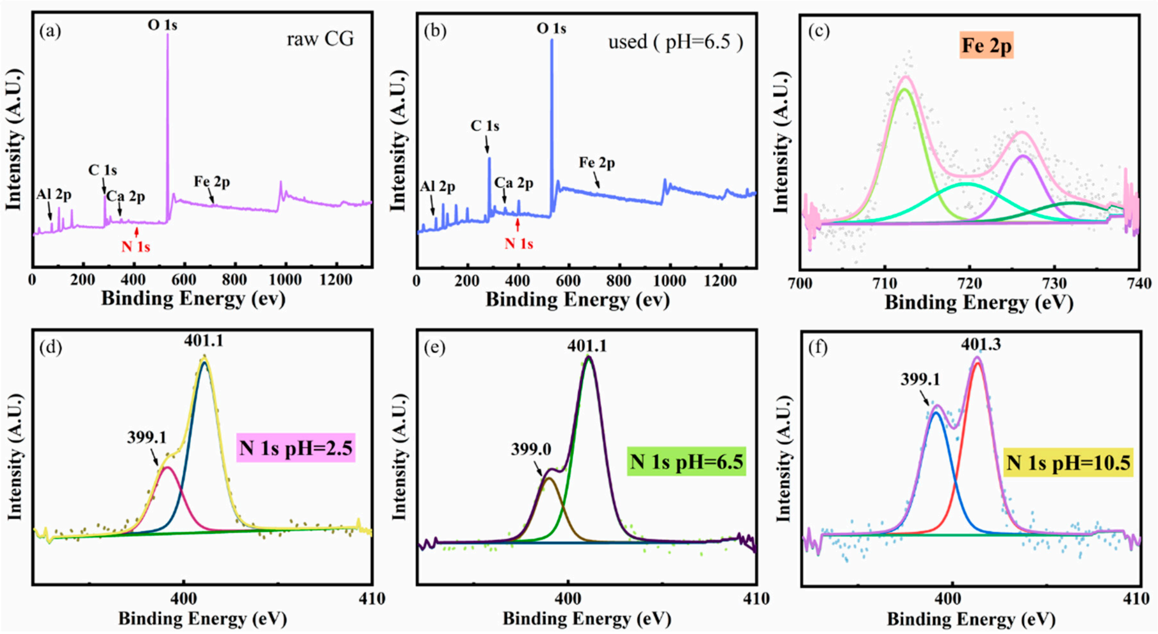

Figure S2. The XPS spectra of the raw and used CG: (a) full-range scan of the raw CG; (b) full-range scan of the used CG (at pH of 6.2); (c) Fe 2p XPS spectra of the raw CG, (d-f) N 1s XPS spectra of the used CG under different pH (pH=2.5, Natural=6.2, pH=10.5). .

The XPS survey spectra confirms the existence of Fe, Ca, and Al in the original CG (Figure S2 a). The peak observed at 712.0 eV was related to the hybrid orbital of Fe 2p3/2 [36, 66]. The peak observed at 726.5 eV was correlated to the Fe 2p3/2 in the CG (Figure S2 c) [67] and the satellite at 719 eV. The peaks at 712.0 eV, 726.5 eV, and 719 eV were related to the valence state of Fe(Ⅲ) in Fe_2O_3, confirming the existence of Fe_2O_3 in CG [67]. The iron and iron oxides in CG revealed its potential for magnetic recovery after TC adsorption process. The XPS spectra of the CG before and after TC removal process at different pHs were measured. The N (400 eV) peaks in Figure S2 b confirmed the adsorption to TC by CG. The peak observed at around 399.0 eV in Figure S2 (d-f) were related to the dimethylamino group [68]. Compared with pH of 2.5 and 6.2, the CG collected after adsorption of TC at pH of 10.5 was possessing increased dimethylamino group, attributed to the TC deprotonation at higher pH condition. At higher pH condition, part of TC^{2-} were adsorbed on the surface of CG.
